# Bidirectional mediation of bone mineral density and brain atrophy on their associations with gait variability

**DOI:** 10.1038/s41598-024-59220-2

**Published:** 2024-04-11

**Authors:** Xin Zhang, Heyang Lu, Min Fan, Weizhong Tian, Yingzhe Wang, Mei Cui, Yanfeng Jiang, Chen Suo, Tiejun Zhang, Li Jin, Kelin Xu, Xingdong Chen

**Affiliations:** 1 School of Public Health, The Key Laboratory of Public Health Safety of Ministry of Education, Fudan University, Shanghai, China; 2 Fudan University Taizhou Institute of Health Sciences, Taizhou, Jiangsu China; 3 Department of Neurology, Huashan Hospital, Fudan University, Shanghai, China; 4 Taixing Disease Control and Prevention Center, Taizhou, Jiangsu China; 5 Taizhou People’s Hospital Affiliated to Nantong University, Taizhou, Jiangsu China; 6 State Key Laboratory of Genetic Engineering, Zhangjiang Fudan International Innovation Center, School of Life Sciences, Human Phenome Institute, Fudan University, Shanghai, China; 7 National Clinical Research Center for Aging and Medicine, Huashan Hospital, Fudan University, Shanghai, China; 8 Yiwu Research Institute of Fudan University, Yiwu, Zhejiang China

**Keywords:** Aging, Brain atrophy, Bone mineral density, Gait, Mediation analysis, Movement disorders, Neurological disorders, Osteoporosis

## Abstract

This mediation analysis aimed to investigate the associations among areal bone mineral density, mobility-related brain atrophy, and specific gait patterns. A total of 595 participants from the Taizhou Imaging Study, who underwent both gait and bone mineral density measurements, were included in this cross-sectional analysis. We used a wearable gait tracking device to collect quantitative gait parameters and then summarized them into independent gait domains with factor analysis. Bone mineral density was measured in the lumbar spine, femoral neck, and total hip using dual-energy X-ray absorptiometry. Magnetic resonance images were obtained on a 3.0-Tesla scanner, and the volumes of brain regions related to mobility were computed using FreeSurfer. Lower bone mineral density was found to be associated with higher gait variability, especially at the site of the lumbar spine (β = 0.174, FDR = 0.001). Besides, higher gait variability was correlated with mobility-related brain atrophy, like the primary motor cortex (β = 0.147, FDR = 0.006), sensorimotor cortex (β = 0.153, FDR = 0.006), and entorhinal cortex (β = 0.106, FDR = 0.043). Bidirectional mediation analysis revealed that regional brain atrophy contributed to higher gait variability through the low lumbar spine bone mineral density (for the primary motor cortex, *P* = 0.018; for the sensorimotor cortex, *P* = 0.010) and the low lumbar spine bone mineral density contributed to higher gait variability through the primary motor and sensorimotor cortices (*P* = 0.026 and 0.010, respectively).

## Introduction

Gait disorder is a common problem among community-dwelling older adults and primarily affects the quality of their lives^1,2^. The nervous and skeletal muscle systems play crucial roles in gait function^3,4^, and brain atrophy and osteoporosis are prevalent in old age. Previous research has shown that volume loss in brain regions responsible for mobility-related functions showed discrepancies in associations with poorer gait performances^5–7^. Specific gait performances have been identified as novel markers for detecting individuals with low bone mineral density (BMD)^8^. Moreover, poor gait performances are related to areal BMD^9,10^. However, the combined impact of mobility-related brain atrophy and low BMD at specific sites on gait remains unclear. Understanding the underlying mechanisms is crucial for developing interventions to enhance gait performance in the aging population.

Many studies found that brain atrophy and low BMD were not wholly independent, with clinical and molecular evidence suggesting biological similarities and interactions between bone and brain^11–13^. The atrophy of the cerebral cortex in Alzheimer's disease patients has been linked to morphometric changes in the cortical microvascular network, where vascular smooth muscle is composed of collagen I^14^. Type I collagen, a key component of bone, is closely related to skeletal abnormalities^15^. Epidemiological evidence indicates a coexistence of brain and bone degeneration in the elderly population. Patients with cognitive impairment, especially those with Alzheimer's disease, are at greater risk for osteoporosis^16^, and BMD continues to decrease as the severity of cognitive impairment increases^17^. A recent Mendelian randomization study also demonstrated a causal relationship between atrophy in different brain regions and low bone density in specific areas^18^. Specifically, the bone–brain axis involves bidirectional communication between the brain and bone tissues. For one thing, neural tissues can produce neurokines that signal down from the brain to bone cells, promoting skeletal homeostasis and regeneration^19^. Animal experiments have proved that proangiogenic factor SLIT3, a contributor to the positive regulation of bone formation, would influence bone mass^20^. But brain atrophy may lead to a reduction in neural factor release. Widespread loss of neurotransmitters has been observed in patients with common neurodegenerative disorders^21^. Such depletion of neural factors may result from the neuronal loss caused by brain atrophy. Meanwhile, osteoporosis, one condition involving inflammation, may affect the survival and activity of osteocytes by inducing the secretion of proinflammatory cytokines and growth factors^22^. Some bone-derived osteokines can cross the blood–brain barrier to reach the central nervous system and affect nerve cells^19^. Lipocalin2 (LCN2), osteopontin (OPN), and insulin-like growth factor 1 (IGF-1) were found to be associated with brain atrophy in a region-specific manner^23–25^. We hypothesize that the reciprocal reinforcement between regional brain atrophy and low BMD might be one mechanism causing specific poor gait performances.

In this population-based study, we investigated the associations of gait performances with low BMD at specific sites and mobility-related brain atrophy. Our study aimed to explore whether brain atrophy and low BMD could mediate each other’s effects on gait performances, shedding light on the potential mechanisms through which bone and brain impact gait.

## Methods

### Study population

This cross-sectional study is based on the Taizhou Imaging Study (TIS), an ongoing community-based cohort study. The inclusion and exclusion criteria for the baseline population have been described in detail in our previous study^26^. Ethical approval for the TIS was obtained from the Ethics Committee of the School of Life Sciences, Fudan University, and Fudan University Taizhou Institute of Health Sciences (Institutional Review Board approval numbers: 496 and B017, respectively). There are two approval numbers because our subjects were recruited in two phases at baseline. Specifically, in phase I, 562 participants (mean age 59.1 years, 46.0% male) were recruited from two villages (Lubao and Hutou). In phase II, 342 subjects (mean age 60.9 years, 42.1% male) were from Caixiang Village. Among the 904 individuals in TIS, 622 participants had complete magnetic resonance imaging (MRI) data, quantitative gait data, and BMD data. After excluding pre-or perimenopausal women and individuals with a history of fracture and thyroid disease, 595 participants, comprising 266 men and 329 postmenopausal women, were finally included in the current analysis. The inclusion and exclusion process is illustrated in Fig. S1. Written informed consent was obtained from all participants before data and biospecimen collection. All methods included in this study were performed in accordance with the Declaration of Helsinki and its later amendments.

### Measurement of gait

A gait tracking device embedded in insoles (Senno gait, Sennotech Co. Ltd., China) was employed to obtain quantitative gait data in multiple dimensions. After placing the insoles comfortably in their shoes, participants were instructed to walk 20 m forth and back in a straight line at their accustomed pace. Vast gait parameters for each stride were recorded and analyzed throughout the procedure, with the gait tracking device automatically excluding the turning strides. The quantitative gait parameters measured included stride time, stance time, swing time, stance time percentage of the gait cycle (%GC) symmetry, swing time (%GC) symmetry, stride time symmetry, stride time coefficient of variation (CV), stance time CV, swing time CV, heel strike angle, stride length, maximum swing velocity, gait velocity, stance time (%GC), and double support time (%GC). Table S1 provides the definitions of each gait parameter and indicates the direction in which a parameter is considered “poorer”. Given that gait velocity is a commonly used measure of overall gait quality, the participants were categorized into two groups based on their gait velocity: the impaired gait group (< 1.0 m/s) and the unimpaired gait group (≥ 1.0 m/s)^27^.

### Measurement of BMD

BMD measurements were conducted at three sites: the lumbar spine, the femoral neck, and the total hip on the left side, using a dual-energy X-ray absorptiometry (Lunar DPX NT-400157; GE Healthcare, Madison, WI, USA). All measurements were assessed by the same experienced operator on the same machine, according to standardized procedures for participant positioning. BMD values were recorded in grams per square centimeter (g/cm^2^). The lumbar spine BMD included the first, second, third, and fourth lumbar vertebra (L1–L4), and the total hip BMD included the whole hip bone. According to the World Health Organization, participants were divided into the normal BMD (T-score ≥ − 1.0), osteopenia (− 1.0 < T-score < − 2.5), or osteoporosis (T-score ≤ − 2.5) groups^28^. These diagnoses were defined with the minimum T-score of three measuring positions.

### MRI acquisition and measurements

MRI scans were conducted at Taizhou People’s Hospital using a 3.0 T scanner (Magnetom Verio Tim scanner; Siemens, Erlangen, Germany). The detailed protocol and sequence parameters for MRI have been previously reported^29^. Based on previous studies, we selected specific brain regions associated with mobility as regions of interest^5,30^. Fifteen mobility-related regions of interest were as follows: motor function (primary motor, sensorimotor), visuospatial attention (inferior posterior parietal lobules, superior posterior parietal lobules), executive control function (dorsolateral prefrontal cortex, anterior cingulate), cognition (hippocampus, entorhinal cortex), motor imagery (precuneus, parahippocampus, posterior cingulated cortex), and balance (pallidum, putamen, caudate, thalamus). FreeSurfer software (v6.0.0) was used to extract these regional volumes from structural T1 images, utilizing the Desikan–Killiany atlas^31^. All brain region volumes were standardized using z-scores for analysis.

### Co-variables

Demographic and lifestyle characteristics data were collected using a questionnaire comprehensively containing age, sex, smoking habits, alcohol and tea consumption, and physical activity. Physical data (height, weight, waist and hip circumferences) were obtained through physical examinations^26^. Waist-to-Hip Ratio (WHR) was calculated by dividing the waist circumference by the hip circumference. Medical history, including hypertension, diabetes, and hyperlipidemia, was defined as the following criteria. Hypertension was defined as a blood pressure greater than 140/90 mmHg, a self-reported history, or current use of antihypertensive drugs. Diabetes was defined as fasting blood glucose greater than 7.0 mmol/L, a self-reported history, or current use of anti-diabetic drugs. Hyperlipidemia was defined as total cholesterol greater than 5.2 mmol/L, triglyceride greater than 1.7 mmol/L, a self-reported history, or current use of antihyperlipidemic drugs.

### Statistical analysis

#### Description analysis

Continuous variables were reported as mean (standard deviation, SD), and categorical variables were presented as frequencies (%). The normality of continuous variables was assessed using the Shapiro–Wilk test, and Levene's test evaluated the homogeneity of variance. Logarithmic transformation was applied to the symmetry gait variables (Fig. S2), whereas square root transformation was applied to the variability gait variables (Fig. S3). Group differences in gait velocity were examined using the following tests: the two-sample t-test or Wilcoxon rank sum test for continuous variables and Pearson's chi-squared test or Fisher's exact test for categorical variables.

#### Factor analysis of quantitative gait parameters

The original variables in the rhythm, symmetry, variability, and phase domains were inverted, representing poorer gait performances with lower values. To extract independent factors with an eigenvalue > 1, fifteen quantitative gait parameters were orthogonally rotated using the “Varimax” rotation method. Gait parameters were then categorized into independent domains based on correlation loadings and the extracted factors. These factors were standardized and expressed as z-scores for further analysis.

### Association analysis

General linear models were employed to investigate the associations among BMD, mobility-related brain regions, and gait performances. The regression analysis, which evaluated the associations of gait performances with BMD and brain volumes, was fitted in two models: Model 1 was adjusted for age, sex, height, and standardized total intracranial volume (included in the analysis of brain volumes); Model 2 was further adjusted for WHR, hypertension, diabetes, hyperlipidemia, tea consumption, smoking, alcohol consumption, and physical activity. A linear trend test was conducted to assess the association between BMD groups and gait domains by scoring the BMD groups from 0 (normal) to 2 (osteoporosis) and entering the score as a continuous term in the regression model. Multiple testing problem was corrected by the false discovery rate (FDR) method.

### Mediation analysis

Two mediation models were fitted with gait performances as the dependent variables for both. In the first set of models, brain atrophy was inserted as the independent variable, with BMD as the candidate mediator. Conversely, in the second set of models, BMD was inserted as the independent variable, while brain atrophy served as the candidate mediator. The mediation analysis was conducted utilizing the R package (mediation) with 1,000 repetitions. Additionally, a sensitivity analysis of mediation was conducted using the R package “medsens” to explore the potential influence of unmeasured confounders on the assumption and assess the robustness of the results, represented as ρ. The threshold of statistical significance was set at adjusted two-tailed *P* < 0.05. Statistical analyses were performed using R software (Version 4.2.2).

### Ethical approval

The studies involving human participants were reviewed and approved by the Ethics Committee of the School of Life Sciences, Fudan University, and Fudan University Taizhou Institute of Health Sciences (institutional review board Approval Number: 496 and B017, respectively). The patients/participants provided their written informed consent to participate in this study.

## Results

### Characteristics of the study population

Table 1 presents the demographic, cardiovascular risk factors, neuroimaging, gait, and BMD characteristics of our participants. The impaired gait group and unimpaired gait group comprised 23.2% and 76.8% of the sample. The two groups differed in sex, age, height, and tea consumption (*P* < 0.05). In addition, participants with impaired gait velocity exhibited smaller gray matter volumes and poorer gait performance, such as maximum swing velocity, heel strike angle, and double support time.

**Table 1:** Characteristics of study participants.

Variable	Study population (n = 595)	Unimpaired gait (n = 457)	Impaired gait (n = 138)	*P*
Demographics				
Female, n (%)	329 (55.3)	240 (52.5)	89 (64.5)	**0.017**
Age, y	59.9 ± 3.1	59.8 ± 3.2	60.5 ± 2.9	**0.015**
Height, cm	159.29 ± 7.84	160.00 ± 7.62	156.96 ± 8.13	** < 0.001**
WHR	0.91 ± 0.06	0.91 ± 0.07	0.91 ± 0.06	0.917
Cardiovascular risk factors				
Current smoker, n (%)	76 (12.8)	64 (14.0)	12 (8.7)	0.134
Alcohol consumption, n (%)	174 (29.2)	142 (31.1)	32 (23.2)	0.093
Tea consumption, n (%)	141 (23.9)	123 (27.0)	18 (13.3)	**0.002**
Physical activity, n (%)	88 (14.8)	72 (15.8)	16 (11.6)	0.285
Hypertension, n (%)	294 (52.5)	218 (50.9)	76 (57.6)	0.216
Hyperlipidemia, n (%)	311 (54.8)	239 (54.6)	72 (55.4)	0.949
Diabetes, n (%)	63 (11.1)	51 (11.7)	12 (9.2)	0.531
Neuroimaging				
Total intracranial volume, cm^3^	1469.92 ± 161.09	1474.72 ± 159.16	1454.03 ± 166.94	0.186
White matter volume, ml	505.89 ± 130.48	506.96 ± 134.00	502.36 ± 118.46	0.717
Gray matter volume, ml	559.15 ± 61.96	561.94 ± 59.85	549.89 ± 67.91	**0.045**
Gait parameters				
Gait velocity,m/s	1.09 ± 0.16	1.15 ± 0.12	0.88 ± 0.12	** < 0.001**
Maximum swing velocity,m/s	3.88 ± 0.55	4.04 ± 0.45	3.32 ± 0.47	** < 0.001**
Heel strike angle, °	7.20 ± 6.72	7.76 ± 6.68	5.34 ± 6.54	** < 0.001**
Double support time (%GC),%	26.35 ± 6.28	26.99 ± 6.23	24.23 ± 5.99	** < 0.001**
BMD, g/cm^2^				
Lumbar spine (L1–L4)	0.97 ± 0.16	0.97 ± 0.16	0.97 ± 0.15	0.920
Femoral neck	0.88 ± 0.13	0.89 ± 0.13	0.87 ± 0.13	0.271
Total hip	0.92 ± 0.14	0.92 ± 0.14	0.91 ± 0.15	0.576
BMD groups, n (%)				
Normal BMD	223 (37.5)	175 (38.3)	48 (34.8)	0.745
Osteopenia	259 (43.5)	197 (43.1)	62 (44.9)	
Osteoporosis	113 (19.0)	85 (18.6)	28 (20.3)	

Categorical variables are presented as numbers (percentages), and continuous variables as means (SDs).

BMD, bone mineral density; WHR, Waist-to-Hip Ratio; %GC, percentage of the gait cycle.

Significant values are in bold.

### Associations between gait domains and different BMD groups

Quantitative gait parameters were summarized into five independent domains with factor analysis, which accounted for 84.70% of the total variance (Table S2). Referring to previous studies^32–34^, we categorized them as rhythm domain (stride time, stance time, swing time), phase domain (double support time percentage of the gait cycle, stance time percentage of the gait cycle), symmetry domain (stance time percentage of the gait cycle symmetry, swing time percentage of the gait cycle symmetry, stride time symmetry), variability domain (stance time coefficient of variation, swing time coefficient of variation, stride time coefficient of variation), and pace domain (stride length, maximum swing velocity, heel strike angle, gait velocity).

**Figure 1:**
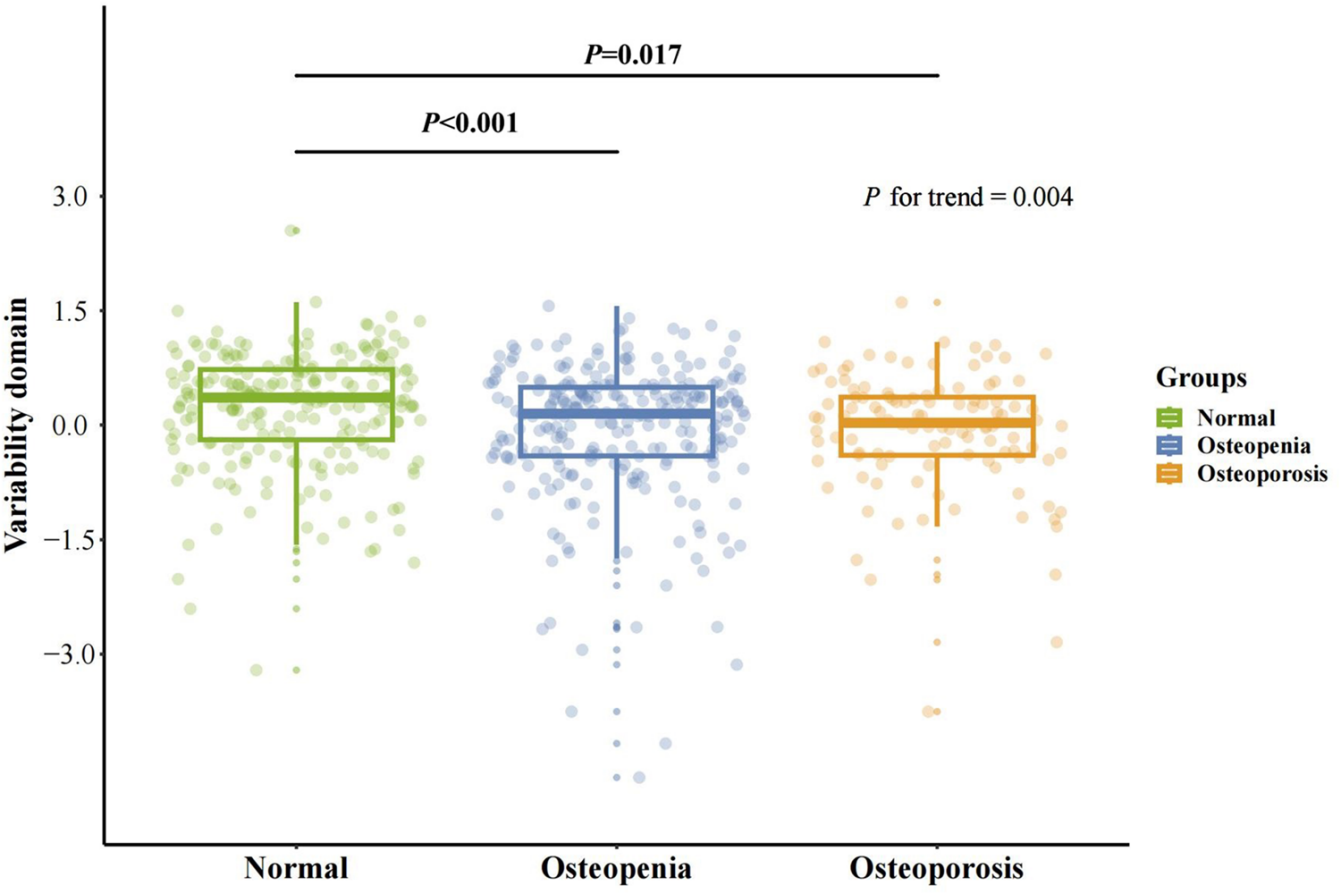
The association between variability domain (z-score) and different BMD groups.

The associations between gait domains and BMD groups were examined (Table S3). Figure 1 shows mean standardized z-scores for gait variability in different groups. After full adjustment (Model 2), we observed a trend (*P* = 0.004) in gait variability across different groups, indicating that participants with lower BMD may exhibit higher variability when walking. However, no statistically significant association was observed in other gait domains, such as rhythm, symmetry, pace, and phase.

### Associations between gait domains and areal BMD

**Figure 2:**
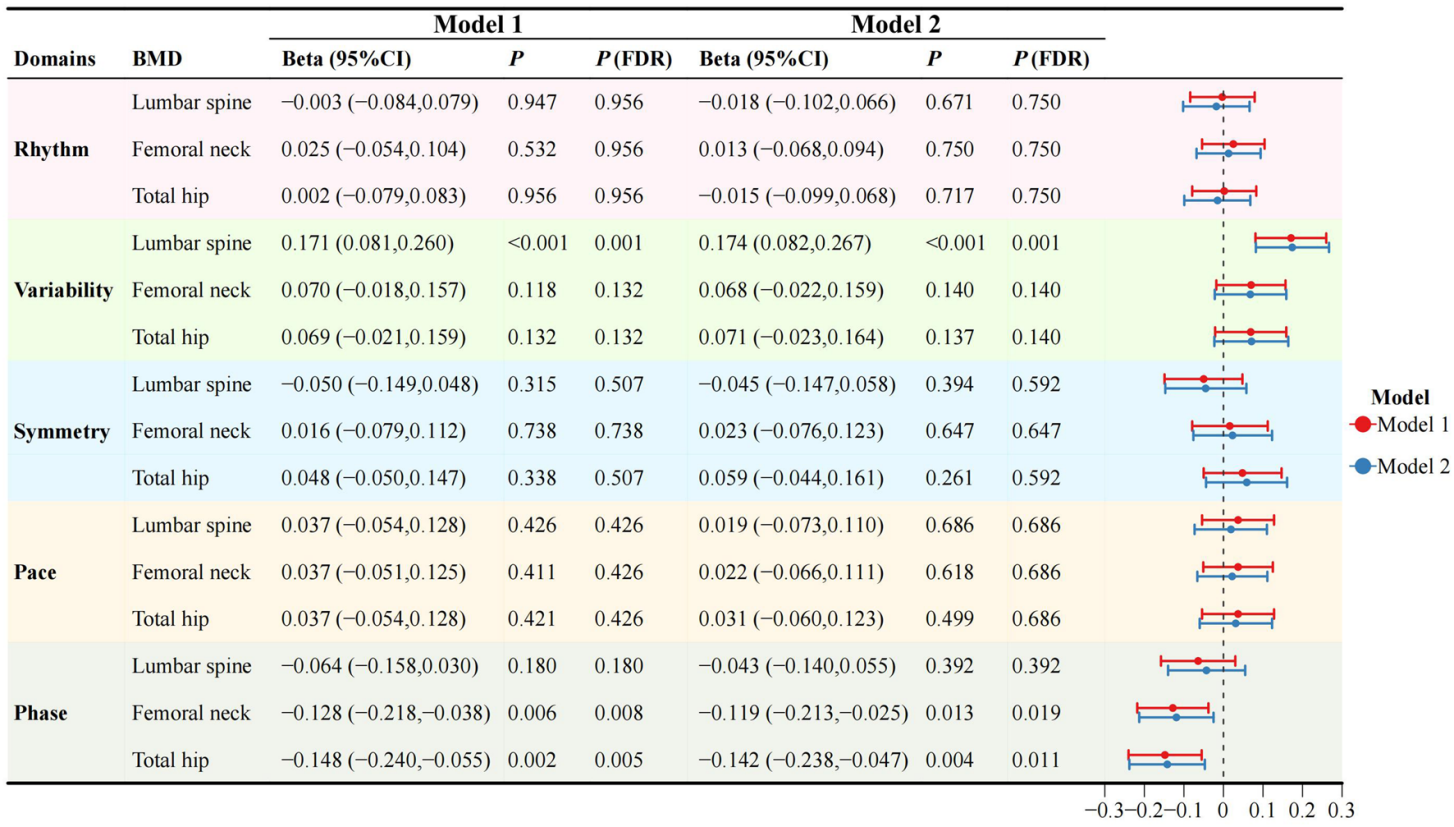
The associations between gait domains and areal BMD.

Considering that different anatomical parts of bones have distinct physiological effects during walking, we further studied the influence of BMD of different anatomical parts on gait variability. Figure 2 shows the associations of various gait domains with areal BMDs (the lumbar spine BMD, the femoral neck BMD, and the total hip BMD) as the independent variables. Lower lumbar spine BMD exhibited a significant association with higher gait variability, a relationship that remained statistically significant even after full adjustment (FDR = 0.001). The negative associations of the phase domain with the femoral neck BMD and the total hip BMD were also significant after correction (both FDR < 0.05).

### Associations between gait domains and mobility-related brain regions

After full adjustment, higher gait variability was significantly associated with smaller volumes in the primary motor (β = 0.147, FDR = 0.006), sensorimotor (β = 0.153, FDR = 0.006), and entorhinal cortex (β = 0.106, FDR = 0.043) (Table 2). Additionally, the pace domain exhibited significant associations with the primary motor (β = 0.263, FDR < 0.001), sensorimotor (β = 0.210, FDR < 0.001), visuospatial attention (β = 0.170, FDR < 0.001), executive control function (β = 0.146, FDR = 0.005), entorhinal cortex (β = 0.155, FDR < 0.001), motor imagery (β = 0.262, FDR < 0.001), and basal ganglia (β = 0.166, FDR = 0.006) regions. Moreover, we also found the associations of the rhythm domain with the primary motor (β = 0.109, FDR = 0.047) and sensorimotor (β = 0.103, FDR = 0.047) cortex were borderline significant after full adjustment (Table S4). However, no statistically significant association was observed between mobility-related brain regions and the symmetry and phase domains (Table S4).

**Table 2:** Associations of mobility-related brain region volumes with gait variability.

Mobility-related regions	Model	Variability		
		β (95%CI)	*P*	*P* (FDR)
Primary motor	Model 1	0.138 (0.056, 0.219)	0.001	**0.004**
	Model 2	0.147 (0.057, 0.236)	0.001	**0.006**
Sensorimotor	Model 1	0.142 (0.058, 0.226)	0.001	**0.004**
	Model 2	0.153 (0.064, 0.242)	0.001	**0.006**
Visuospatial attention	Model 1	0.044 (− 0.042, 0.130)	0.313	0.542
	Model 2	0.032 (− 0.060, 0.124)	0.491	0.786
Executive control	Model 1	0.022 (− 0.075, 0.120)	0.652	0.745
	Model 2	0.007 (− 0.095, 0.109)	0.887	0.901
Hippocampus	Model 1	0.005 (− 0.097, 0.106)	0.929	0.929
	Model 2	0.007 (− 0.096, 0.109)	0.901	0.901
Entorhinal cortex	Model 1	0.096 (0.016, 0.177)	0.019	0.052
	Model 2	0.106 (0.020, 0.192)	0.016	**0.043**
Motor imagery	Model 1	0.041 (− 0.044, 0.127)	0.345	0.542
	Model 2	0.034 (− 0.059, 0.127)	0.476	0.786
Basal ganglia	Model 1	0.048 (− 0.066, 0.163)	0.406	0.542
	Model 2	0.017 (− 0.103, 0.138)	0.777	0.901

Values are estimated coefficients (95%CI). Model 1 was adjusted for sex, age, height, and standardized total intracranial volume (TIV); Model 2 was further adjusted for WHR, hypertension, diabetes, hyperlipidemia, smoking, alcohol consumption, and physical activity.

WHR, Waist-to-Hip Ratio; FDR, false discovery rate.

Significant values are in bold.

### Mediation analysis

**Figure 3:**
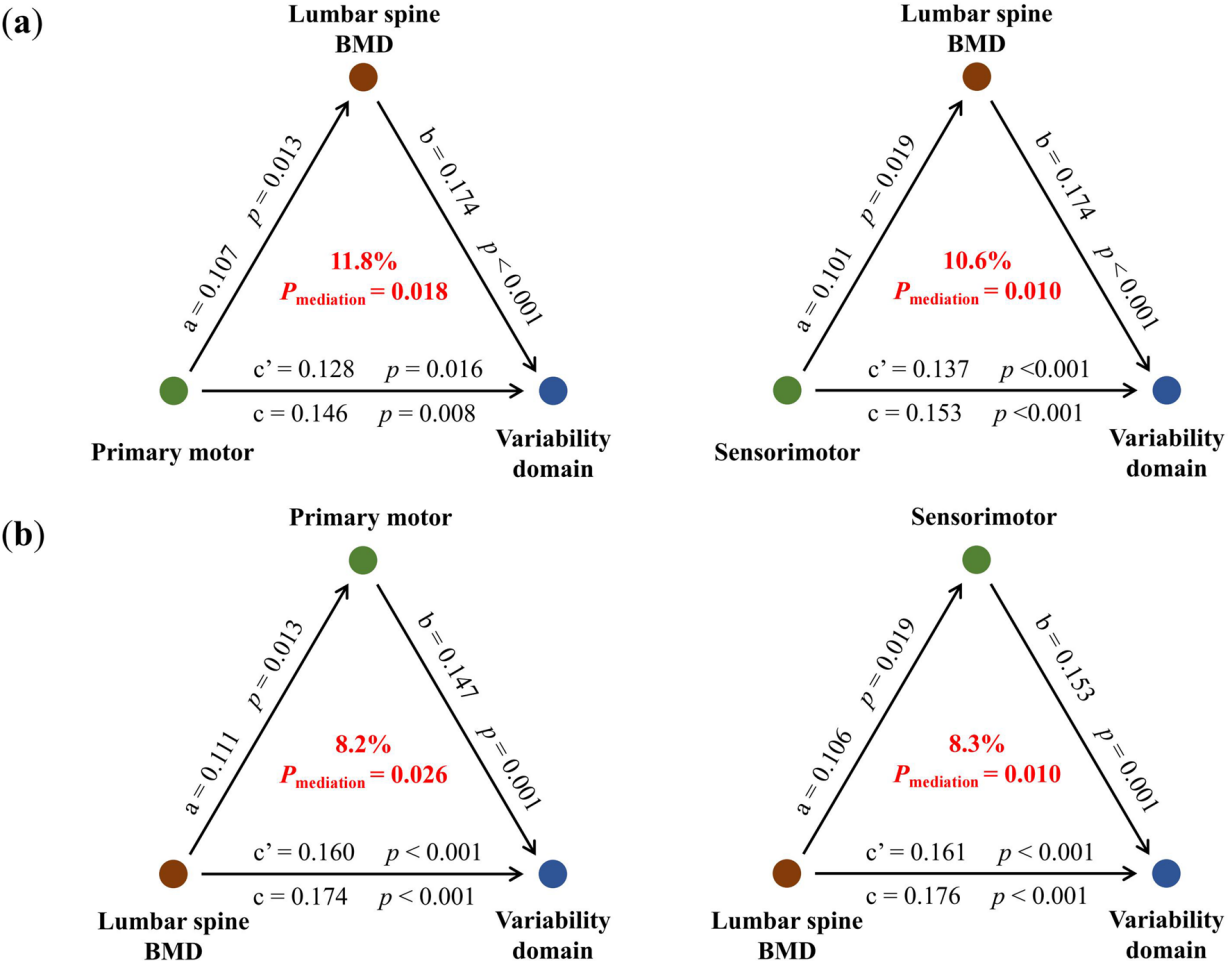
Mediation linkages among the lumbar spine BMD, mobility-related brain atrophy, and gait variability.

Bidirectional mediation analysis was conducted to assess the combined impact of lumbar spine BMD and mobility-related brain atrophy on gait variability. The findings indicated that the atrophy of mobility-related brain regions, such as the primary motor cortex and sensorimotor cortex, may contribute to higher gait variability by decreasing the lumbar spine BMD (Fig. 3a). Specifically, the lumbar spine BMD explained 11.8% of the total effect of the primary motor cortex on gait variability (*P*_mediation_ = 0.018) and 10.6% of the total effect of the sensorimotor cortex on gait variability (*P*_mediation_ = 0.010). Furthermore, the reverse mediation analyses revealed that the lumbar spine BMD could also influence the gait variability through the two brain regions (Fig. 3b). The mediating effect of the primary motor cortex represented 8.2% (*P*_mediation_ = 0.026), while that of the sensorimotor cortex accounted for 8.3% (*P*_mediation_ = 0.010). Additional detailed information was provided in Tables S5 and S6.

### Sensitivity analysis

We conducted sensitivity analyses to assess the potential impact of unmeasured confounding. The results in Tables S7 and S8 indicated that the estimated indirect effects were relatively robust to unmeasured confounders, as indicated by the sensitivity parameter (ρ).

## Discussion

This study found evidence for a bidirectional relationship between BMD and brain atrophy concerning gait variability. Specifically, we found that the lumbar spine BMD partially mediated the effects of the primary motor cortex and sensorimotor cortex on gait variability, and the primary motor cortex and the sensorimotor cortex partially mediated the effect of the lumbar spine BMD on gait variability. Although our results showed a small-to-medium range of effect sizes, we consider it reasonable because the role of a single anatomical site of the brain and bone is limited.

Previous studies have indicated a link between brain atrophy and gait variability. Specifically, a meta-analysis of 25 studies found a strong association between high gait variability and neurodegeneration^35^. Compared to other gait domains, variability can help to differentiate Alzheimer's disease dementia, a common neurodegenerative disorder, which can be observed atrophy in specific brain regions^36^. Notably, higher step length variability is associated with the atrophy of the hippocampus, superior parietal lobe, and anterior cingulate gyrus, regions responsible for cognition and executive control that matter in mobility^7^. Similarly, our research has corroborated these findings, showing that brain atrophy affecting motor function and cognition, including areas like the primary motor cortex, sensorimotor cortex, and entorhinal cortex, is associated with higher gait variability. However, to our knowledge, no existing studies have investigated the potential impact of brain atrophy on gait performance through BMD. The following evidence supports our finding that the lumbar spine BMD can explain the effect of mobility-related brain atrophy on gait variability. First, higher gait variability seems to be a shared gait performance between individuals with brain atrophy and those with lower BMD. Besides, based on the bone–brain axis, brain atrophy may directly affect central control of bone remodeling, thus resulting in low BMD^37^. A recent Mendelian randomization study has further supported these relationships by establishing causal links between the atrophy of specific brain regions and BMD at distinct sites^18^. Our results provided evidence of the mediating effect of BMD on the association between brain atrophy and gait.

Likewise, the potential mediating role of brain atrophy between BMD and gait performance has not been studied. Previous studies have indicated an association between low BMD and mobility impairment, such as balance, variability, and symmetry^38–40^. This may be attributed to less propulsion and stability caused by abnormal changes in kinematics and kinetics parameters of the trunk or extremities^41^. Such associations between specific physical performances and areal BMD were informed before^10,42^. Similarly, we found a significant association between low lumbar spine BMD and higher gait variability and the mediating role of mobility-related brain atrophy. Although there are no direct comparisons available for our specific findings, existing evidence indirectly supports our findings. First, some elderly-based studies have observed that individuals with osteoporosis tend to exhibit greater parenchymal atrophy and significant ventricular enlargement and that BMD loss was correlated with regional gray matter volume decline^43,44^. Second, patients with osteoporotic vertebral compression fractures were found to have smaller volumes of brain parenchyma^45^, which emphasizes the association of the lumbar spine BMD with brain atrophy. Finally, insights from the bone–brain axis suggest that osteokines, like IGF-1, could help improve behavioral phenotypes in mouse models^46^ and have also been associated with gait speed in older adults^47^.

Lumbar BMD was observed to have a more substantial impact on gait variability compared to femoral neck and total hip BMD. This is likely attributed to several factors. Firstly, potential vertebral compression fractures could be a contributing factor. Patients suffering from vertebral compression fractures may experience varying degrees of lower back pain, resulting in reduced lumbar extensor function and increased gait variability^48^. Lumbar acceleration was also identified as being more indicative of gait stability. In terms of biomechanics, the stability of the human body during walking primarily depends on the ability to regulate the movement of the center of mass (COM)^49^, typically situated at the junction of the waist and pelvis of the human body^50^.

Our study contributes to the existing literature by examining and providing evidence for the bidirectional relationship between areal BMD, regional brain atrophy, and their combined effects on gait variability in elderly adults. To enhance the sensitivity of gait assessments, we employed a quantitative approach using an insole-style wearable gait tracking device, addressing limitations of commonly used qualitative or semi-quantitative methods. The gait parameters collected were categorized into distinct domains representing specific gait patterns in our study. Gait variability assessment can serve as a valuable tool for potentially identifying elderly individuals with low lumbar BMD or reduced motor cortex volume. By detecting these conditions early, interventions targeting bone and brain degeneration processes can be initiated, ultimately delaying their progression and promoting increased years of healthy living. Moreover, gait variability assessment can also play a role in evaluating the efficacy of bone and brain health care for patients, offering valuable insights to guide treatment strategies and rehabilitation efforts to enhance physical function.

However, our results should be interpreted in light of several limitations. First, causal conclusions cannot be drawn in this cross-sectional study. Further investigation using longitudinal designs of TIS would be valuable to help rectify this limitation. Second, our sample is a Chinese rural population around 60 years old, potentially limiting the generalizability of our findings to other population groups. Future research could involve individuals in our expanded cohort to enhance external validity. Third, potential back pain resulting from vertebral compression fractures was not taken into account^51,52^. In the future, we will consider adding a measurement of vertebral compression fractures and related pain severity when conducting the DXA examination. Finally, muscular factors were not considered in the present study. Including these factors in future studies would provide a more comprehensive understanding of gait.

## Conclusion

The present study indicates that brain atrophy, especially mobility-related brain regions, and low BMD in the lumbar spine are associated with higher gait variability. Importantly, bidirectional mediation analysis conducted for the first time identifies the interrelationship between mobility-related brain atrophy and low lumbar spine BMD in gait variability. However, additional extensive population studies and longitudinal investigations are required to validate our findings and provide a more comprehensive understanding of the effects of brain and bone on gait changes in more detail.

### Supplementary Information

Supplementary Information.

## Data Availability

The datasets used and/or analyzed in this study are available from the corresponding author on reasonable request.
