# Supplementary material for: Bidirectional mediation of bone mineral density and brain atrophy on their associations with gait variability
Source: Sci Rep. 2024 Apr 11;14:8483. doi: 10.1038/s41598-024-59220-2 (PMC11009386; doi:10.1038/s41598-024-59220-2)
Supplement: Supplementary file 1 — Supplementary Information. [file 41598_2024_59220_MOESM1_ESM.pdf]

Bidirectional mediation of bone mineral density and brain atrophy on their associations with gait variability

Xin Zhang[1,2], Heyang Lu[3], Min Fan[4], Weizhong Tian[5], Yingzhe Wang[2,6], Mei Cui[2,3], Yanfeng Jiang[2,6], Chen Suo[1,2], Tiejun Zhang[1,2], Li Jin[2,6], Kelin Xu[1,2,*], and Xingdong Chen[2,6,7,8,*]

[1]School of Public Health, the Key Laboratory of Public Health Safety of Ministry of Education, Fudan University, Shanghai, China

[2]Fudan University Taizhou Institute of Health Sciences, Taizhou, Jiangsu, China

[3]Department of Neurology, Huashan Hospital, Fudan University, Shanghai, China

[4]Taixing Disease Control and Prevention Center, Taizhou, Jiangsu, China

[5]Taizhou People's Hospital Affiliated to Nantong University, Taizhou, Jiangsu, China

[6]State Key Laboratory of Genetic Engineering, Zhangjiang Fudan International Innovation Center, School of Life Sciences, Human Phenome Institute, Fudan University, Shanghai, China

[7]National Clinical Research Center for Aging and Medicine, Huashan Hospital, Fudan University, Shanghai, China

[8]Yiwu Research Institute of Fudan University, Yiwu, Zhejiang, China

*Address correspondence to: Kelin Xu. Email: xukelin@fudan.edu.cn; Xingdong Chen. Email: xingdongchen@fudan.edu.cn.

Catalogue

Supplementary Materials 3

Supplementary Tables........ 3

Table S1. Definition of gait parameters. 3
Table S2. Factor analysis of gait parameters........ 4
Table S3. Associations of osteopenia and osteoporosis with gait domains........ 5
Table S4. Associations of mobility-related brain region volumes with gait domains. 6
Table S5. The model for mediating effect of lumbar spine BMD on the association between brain structure and variability domain. 8
Table S6. The model for mediating effect of brain structure on the association between lumbar spine BMD and variability domain. 9
Table S7. Sensitivity analysis of the mediation effect of lumbar spine BMD in the association of brain atrophy and variability domain. 10

Supplementary Figures 12

Fig S1. The flowchart of participants in this study. 12
Fig S2. Logarithmic transformation for the symmetry gait variables. 13
Fig S3. Square root transformation for the variability gait variables. 14

Supplementary Materials

Supplementary Tables

Table S1. Definition of gait parameters.

Parameter	Description	Indication of "poorer" gait	Domain
Stride time	The time between the first contacts of two consecutive footfalls of the same foot.	Higher	Rhythm
Stance time	The time of the cycle during which part of the foot touches the ground. It begins when the foot first touches the ground and ends when the same foot leaves the ground.	Higher	Rhythm
Swing time	The time of the cycle during which the foot is in the air and does not touch the ground. It begins when the foot first leaves the ground and ends when the same foot touches the ground again.	Higher	Rhythm
Stance time %GC symmetry	The ratio of the higher stance time %GC to the lower.	Higher	Symmetry
Swing time %GC symmetry	The ratio of the higher swing time %GC to the lower.	Higher	Symmetry
Stride time symmetry	The ratio of the higher stride time to the lower.	Higher	Symmetry
Stance time CV	The coefficient of variation of stance time.	Higher	Variability
Swing time CV	The coefficient of variation of swing time.	Higher	Variability
Stride time CV	The coefficient of variation of stride time.	Higher	Variability
Heel strike angle	The angle between the foot and the ground at heel strike, on a vertical plane.	Lower	Pace
Stride length	The distance between two consecutive footprints on the ground, parallel to the line of progression.	Lower	Pace
Maximum swing velocity	The maximum forward speed of the foot during swing.	Lower	Pace
Gait velocity	The mean speed of forward walking, calculated in meters per second.	Lower	Pace
Stance time (%GC)	The percentage of the stance time in the gait cycle.	Higher	Phase
Double support time (%GC)	The percentage of the double support time in the gait cycle.	Higher	Phase

Abbreviation: CV, coefficient of variation; %GC, percentage of the gait cycle.

Table S2. Factor analysis of gait parameters.

Gait Parameter	**Rhythm**	**Variability**	**Symmetry**	**Pace**	**Phase**
**Rhythm**					
Stride time, ms	**0.924**	0.233	0.213	0.105	0.093
Stance time, ms	**0.874**	0.250	0.053	0.102	0.347
Swing time, ms	**0.773**	-0.109	0.320	0.054	-0.453
**Variability**					
Swing time CV, %	0.115	**0.879**	0.278	0.173	0.153
Stance time CV, %	0.115	**0.879**	0.278	0.173	0.153
Stride time CV, %	0.136	**0.775**	0.265	0.167	-0.015
**Symmetry**					
Stance time symmetry	0.117	0.203	**0.939**	0.091	-0.006
Swing time symmetry	0.118	0.318	**0.861**	0.098	0.108
Stride time symmetry	0.250	0.362	**0.753**	0.083	-0.058
**Pace**					
Stride length, m	0.145	0.210	0.106	**0.784**	0.430
Heel strike angle, °	-0.168	0.253	0.089	**0.781**	-0.144
Maximum swing velocity, m/s	0.534	0.101	0.075	**0.762**	0.158
Gait velocity, m/s	0.485	0.055	0.105	**0.623**	0.405
**Phase**					
Stance time (%GC), %	0.227	0.271	-0.207	0.073	**0.835**
Double support time (%GC), %	0.025	0.028	-0.167	-0.143	**-0.687**
**% of variance**	40.79	15.93	13.12	8.55	6.31

Note: The highest loading variables are highlighted in bold.

Abbreviation: CV, coefficient of variation; %GC, percentage of the gait cycle.

Table S3. Associations of osteopenia and osteoporosis with gait domains.

Domains	Model	Normal BMD	Osteopenia		Osteoporosis		*P* for trend
			**β (95% CI)**	***P***	**β (95% CI)**	***P***	
Rhythm	Model 1	Reference	-0.009 (-0.180,0.162)	0.920	-0.031 (-0.262,0.200)	0.789	0.799
	Model 2	Reference	0.011 (-0.163,0.185)	0.901	-0.033 (-0.269,0.203)	0.784	0.829
Variability	Model 1	Reference	-0.357 (-0.544,-0.169)	**<0.001**	-0.330 (-0.583,-0.076)	**0.011**	**0.003**
	Model 2	Reference	-0.374 (-0.566,-0.182)	**<0.001**	-0.318 (-0.579,-0.057)	**0.017**	**0.004**
Symmetry	Model 1	Reference	0.055 (-0.151,0.262)	0.600	0.175 (-0.104,0.454)	0.219	0.236
	Model 2	Reference	0.047 (-0.167,0.260)	0.667	0.167 (-0.123,0.457)	0.258	0.280
Pace	Model 1	Reference	-0.033 (-0.225,0.158)	0.735	-0.098 (-0.357,0.160)	0.455	0.468
	Model 2	Reference	-0.004 (-0.194,0.187)	0.970	-0.053 (-0.312,0.205)	0.686	0.716
Phase	Model 1	Reference	-0.061 (-0.258,0.136)	0.542	0.209 (-0.057,0.474)	0.124	0.223
	Model 2	Reference	-0.087 (-0.288,0.115)	0.400	0.177 (-0.097,0.451)	0.206	0.348

Notes: Model 1 was adjusted for sex, age, and height; Model 2 was further adjusted for WHR, hypertension, diabetes, hyperlipidemia, tea consumption, smoking, alcohol consumption, and physical activity.

Table S4. Associations of mobility-related brain region volumes with gait domains.

Mobility-related regions	Model	Rhythm			Symmetry			Pace			Phase		
		**β (95%CI)**	***P***	***P* (FDR)**	**β (95%CI)**	***P***	***P* (FDR)**	**β (95%CI)**	***P***	***P* (FDR)**	**β (95%CI)**	***P***	***P* (FDR)**
Primary motor	Model 1	0.058 (-0.016,-0.132)	0.121	0.486	0.093 (0.003,0.182)	0.043	0.171	0.303 (0.224,0.382)	<0.001	**<0.001**	-0.029 (-0.115,0.057)	0.502	0.670
	Model 2	0.109 (0.028,0.189)	0.008	**0.047**	0.075 (-0.024,0.174)	0.139	0.472	0.263 (0.178,0.349)	<0.001	**<0.001**	0.003 (-0.091,0.098)	0.945	0.945
Sensorimotor	Model 1	0.070 (-0.006,0.146)	0.072	0.486	0.084 (-0.008,0.176)	0.074	0.174	0.263 (0.181,0.345)	<0.001	**<0.001**	-0.003 (-0.091,0.085)	0.946	0.946
	Model 2	0.103 (0.023,0.183)	0.012	**0.047**	0.065 (-0.034,0.163)	0.200	0.472	0.210 (0.124,0.296)	<0.001	**<0.001**	0.022 (-0.073,0.116)	0.650	0.945
Visuospatial attention	Model 1	0.038 (-0.039,0.114)	0.337	0.539	0.081 (-0.012,0.174)	0.087	0.174	0.215 (0.132,0.299)	<0.001	**<0.001**	-0.045 (-0.134,0.044)	0.322	0.670
	Model 2	0.072 (-0.009,0.154)	0.082	0.154	0.057 (-0.043,0.157)	0.265	0.472	0.170 (0.082,0.258)	<0.001	**<0.001**	-0.024 (-0.120,0.072)	0.626	0.945
Executive control function	Model 1	0.046 (-0.041,0.133)	0.300	0.539	0.040 (-0.066,0.146)	0.460	0.526	0.183 (0.087,0.280)	<0.001	**<0.001**	-0.041 (-0.142,0.060)	0.423	0.670
	Model 2	0.073 (-0.018,0.163)	0.115	0.154	0.029 (-0.083,0.140)	0.610	0.698	0.146 (0.047,0.244)	0.004	**0.005**	-0.025 (-0.131,0.082)	0.648	0.945
Hippocampus	Model 1	0.059 (-0.032,0.150)	0.206	0.539	-0.056 (-0.166,0.054)	0.318	0.424	-0.041 (-0.142,0.060)	0.428	0.428	-0.086 (-0.191,0.019)	0.108	0.670
	Model 2	0.044 (-0.047,0.136)	0.342	0.391	-0.055 (-0.167,0.058)	0.341	0.472	-0.033 (-0.133,0.067)	0.523	0.523	-0.093 (-0.200,0.014)	0.088	0.708
Entorhinal cortex	Model 1	0.014 (-0.059,0.087)	0.709	0.709	0.110 (0.023,0.198)	0.014	0.110	0.200 (0.121,0.279)	<0.001	**<0.001**	-0.029 (-0.113,0.056)	0.505	0.670
	Model 2	0.101 (0.007,0.195)	0.035	0.094	0.101 (0.007,0.195)	0.035	0.283	0.155 (0.072,0.238)	<0.001	**<0.001**	-0.010 (-0.100,0.080)	0.832	0.945
Motor imagery	Model 1	0.031 (-0.046,0.108)	0.428	0.571	0.069 (-0.024,0.162)	0.145	0.231	0.299 (0.218,0.381)	<0.001	**<0.001**	-0.025 (-0.113,0.064)	0.586	0.670

	Model 2	0.069 (-0.014,0.152)	0.101	0.154	0.048 (-0.054,0.150)	0.354	0.472	0.262 (0.174,0.349)	<0.001	**<0.001**	-0.005 (-0.103,0.092)	0.913	0.945
Basal ganglia	Model 1	-0.020 (-0.123,0.082)	0.697	0.709	0.022 (-0.102,0.146)	0.730	0.730	0.254 (0.141,0.366)	<0.001	**<0.001**	-0.070 (-0.189,0.049)	0.249	0.670
	Model 2	0.000 (-0.107,0.108)	0.999	0.999	0.007 (-0.125,0.138)	0.920	0.920	0.166 (0.050,0.282)	0.005	**0.006**	-0.015 (-0.140,0.111)	0.818	0.945

Notes: Values are estimated coefficients (95%CI). Model 1 was adjusted for sex, age, height, and standardized TIV; Model 2 was further adjusted for WHR, hypertension, diabetes, hyperlipidemia, tea consumption, smoking, alcohol consumption, and physical activity.
Abbreviations: FDR, false discovery rate.

Table S5. The model for mediating effect of lumbar spine BMD on the association between brain structure and variability domain.

	**Primary motor**		**Sensorimotor**		**Entorhinal cortex**	
	**Estimate (95%CI)**	***P***	**Estimate (95%CI)**	***P***	**Estimate (95%CI)**	***P***
Total effect (c)	0.146 (0.042,0.260)	0.008	0.153 (0.054,0.260)	<0.001	0.105 (0.005,0.220)	0.034
Direct effect (c’)	0.128 (0.026,0.240)	0.016	0.137 (0.039,0.240)	<0.001	0.099 (0.002,0.220)	0.044
Path a (X on M)	0.107 (0.023,0.192)	0.013	0.101 (0.017,0.185)	0.019	0.031 (-0.050,0.112)	0.450
Path b (M on Y)	0.174 (0.082,0.267)	<0.001	0.174 (0.082,0.267)	<0.001	0.174 (0.082,0.267)	<0.001
Indirect effect (ab)	0.017 (0.004,0.030)	0.010	0.016 (0.004,0.030)	0.010	0.005 (-0.010,0.020)	0.448
Proportion mediated	0.118 (0.024,0.440)	0.018	0.106 (0.024,0.330)	0.010	0.051 (-0.209,0.390)	0.462

Table S6. The model for mediating effect of brain structure on the association between lumbar spine BMD and variability domain.

	Primary motor		Sensorimotor		Entorhinal cortex	
	**Estimate (95%CI)**	***P***	**Estimate (95%CI)**	***P***	**Estimate (95%CI)**	***P***
Total effect (c)	0.174 (0.093,0.260)	<0.001	0.176 (0.095,0.260)	<0.001	0.175 (0.095,0.260)	<0.001
Direct effect (c’)	0.160 (0.078,0.240)	<0.001	0.161 (0.079,0.240)	<0.001	0.171 (0.090,0.250)	<0.001
Path a (X on M)	0.111 (0.024,0.199)	0.013	0.106 (0.018,0.194)	0.019	0.036 (-0.057,0.128)	0.450
Path b (M on Y)	0.147 (0.057,0.236)	0.001	0.153 (0.064, 0.242)	0.001	0.106 (0.020, 0.192)	0.016
Indirect effect (ab)	0.014 (0.001,0.040)	0.026	0.015 (0.002,0.030)	0.010	0.004 (-0.007,0.020)	0.480
Proportion mediated	0.082 (0.009,0.230)	0.026	0.083 (0.010,0.220)	0.010	0.020 (-0.042,0.100)	0.480

Table S7. Sensitivity analysis of the mediation effect of lumbar spine BMD in the association of brain atrophy and variability domain.

Sensitivity results	Outcome
	**Variability**
**Primary motor**	
For lumbar spine BMD: ρ at which ACME = 0	0.149
**Sensorimotor**	
For lumbar spine BMD: ρ at which ACME = 0	0.151

Table S8. Sensitivity analysis of the mediation effect of brain atrophy in the association of lumbar spine BMD and variability domain.

Sensitivity results	Outcome
	**Variability**
**Lumbar spine BMD**	
For primary motor: ρ at which ACME = 0	0.123
For sensorimotor: ρ at which ACME = 0	0.132

Supplementary Figures

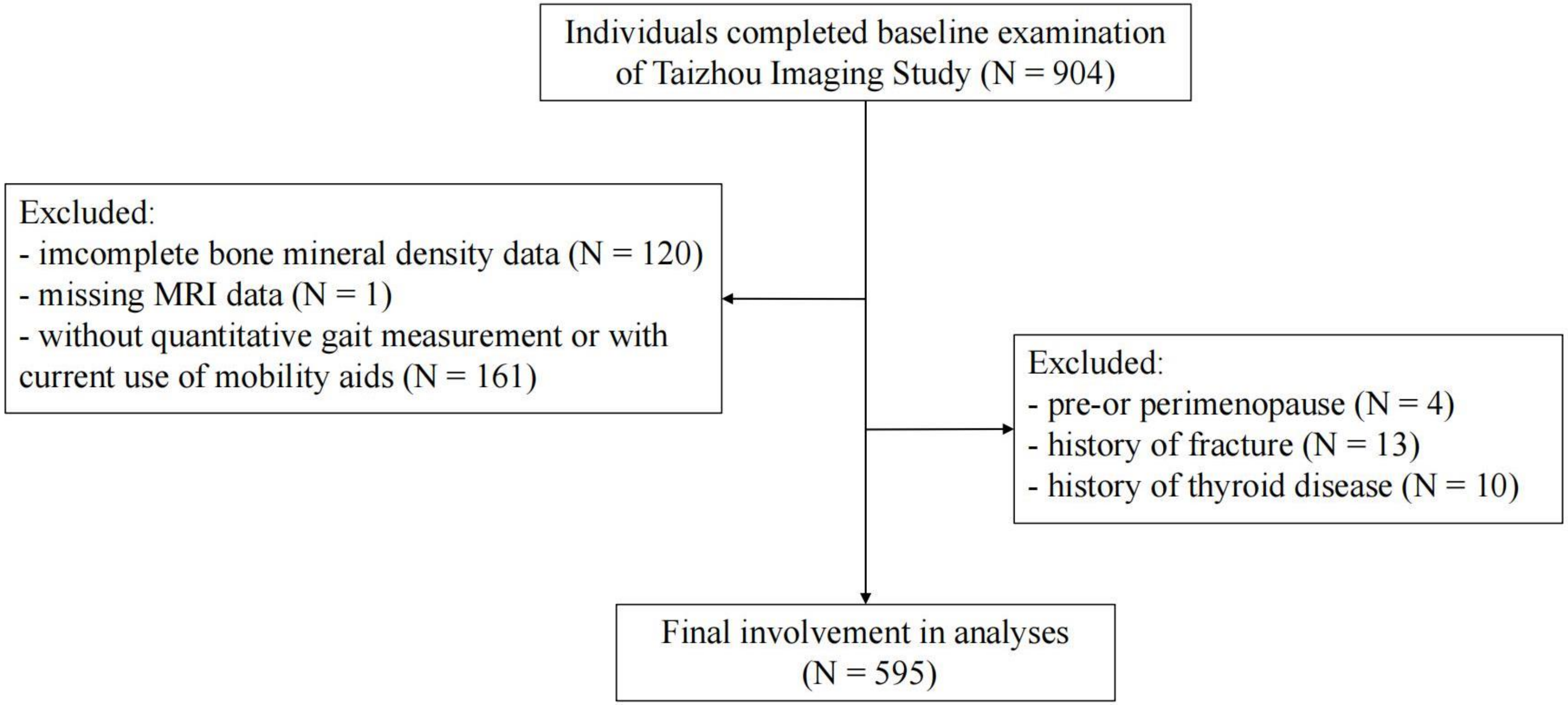

Fig S1. The flowchart of participants in this study.

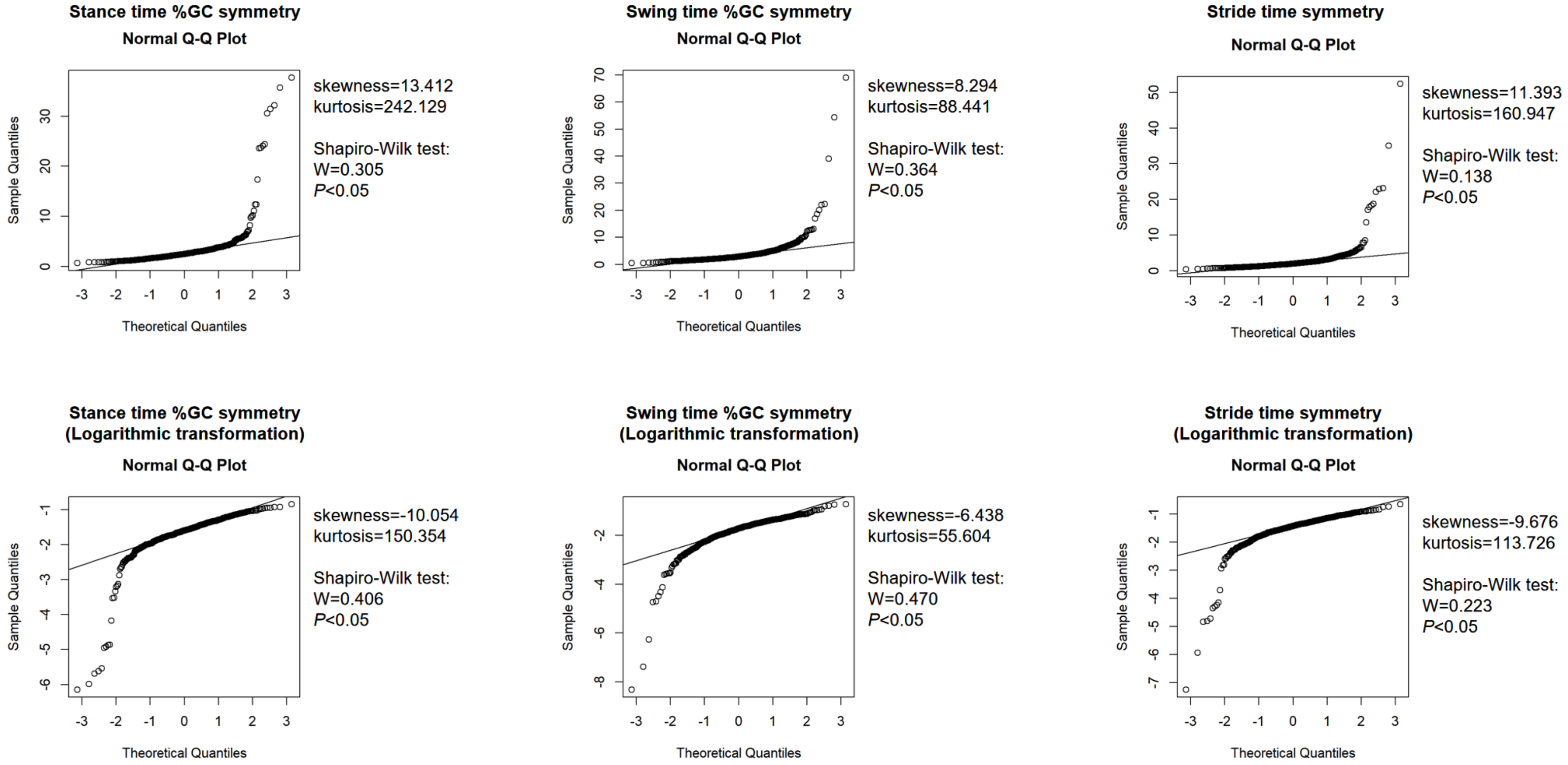

Fig S2. Logarithmic transformation for the symmetry gait variables.

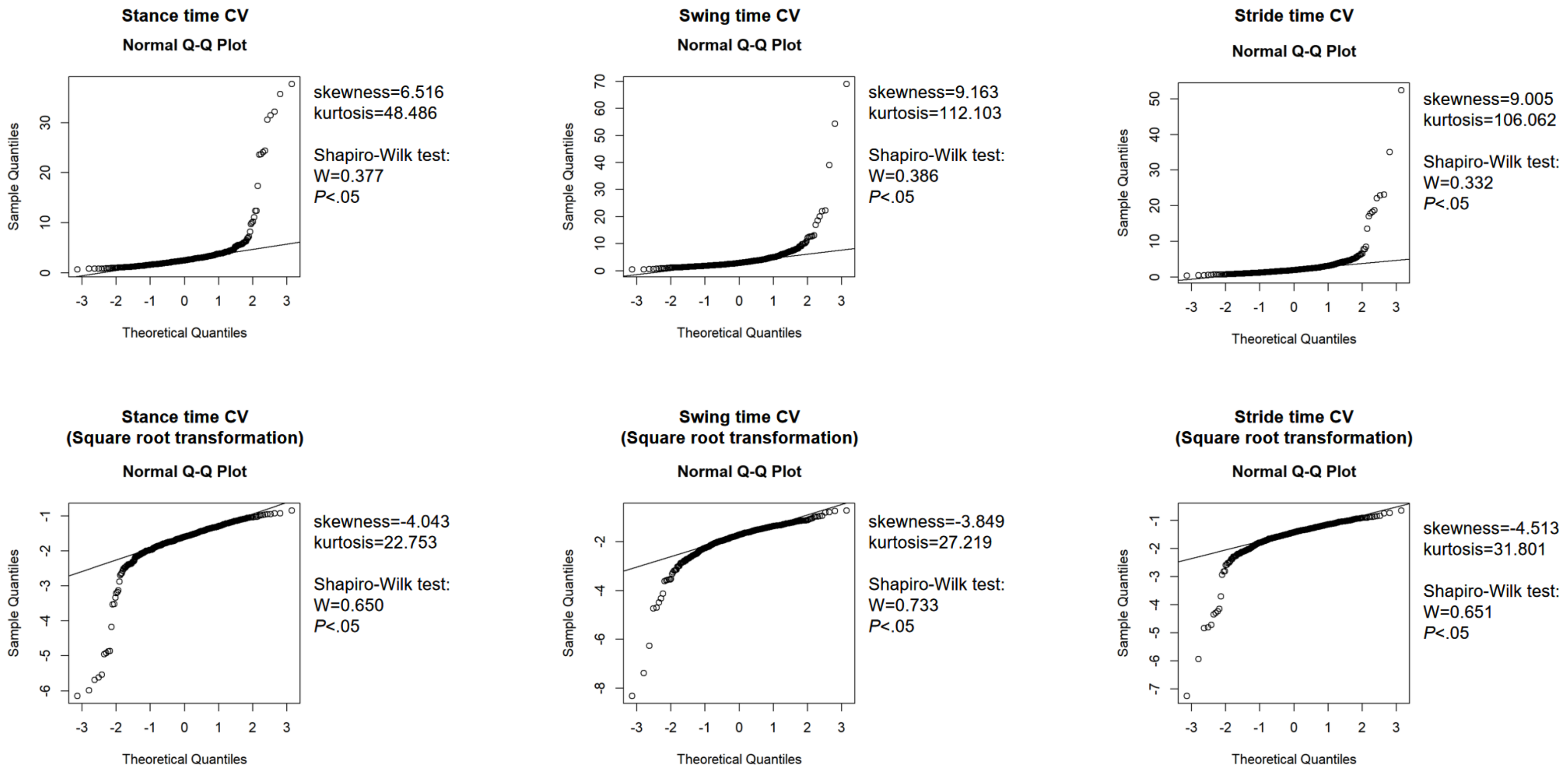

Fig S3. Square root transformation for the variability gait variables.
